# Risks of Adverse Events Following Coprescription of Statins and Calcium Channel Blockers: A Nationwide Population-Based Study

**DOI:** 10.1097/MD.0000000000002487

**Published:** 2016-01-15

**Authors:** Yi-Chun Wang, Tsung-Cheng Hsieh, Chu-Lin Chou, Jung-Lun Wu, Te-Chao Fang

**Affiliations:** From the Institute of Medical Sciences, Tzu Chi University, Hualien (Y-CW, T-CH); Division of Nephrology, Taipei Tzu Chi Hospital, Buddhist Tzu Chi Medical Foundation, Taipei (Y-CW); School of Medicine, Tzu Chi University, Hualien (Y-CW); Division of Nephrology, Department of Internal Medicine, Tri-Service General Hospital, National Defense Medical Center, Taipei (C-LC); Department of Occupational Medicine, Hualien Tzu Chi Hospital, Buddhist Tzu Chi Medical Foundation, Hualien (J-LW); Division of Nephrology, Department of Internal Medicine, Wan Fang Hospital, Taipei Medical University, Taipei (T-CF); and Department of Internal Medicine, School of Medicine, College of Medicine, Taipei Medical University, Taipei, Taiwan (T-CF).

## Abstract

Some statins (simvastatin, lovastatin, and atorvastatin) are metabolized by cytochrome P450s 3A4 (CYP3A4). Inhibitors of CYP3A4 including some calcium channel blockers (CCBs) might increase statin blood concentration, owing to drug–drug interactions. Risk of adverse events such as acute kidney injury might occur following the coprescription of CYP3A4-metabolized statins and CCBs that inhibit CYP3A4.

This was a population-based cohort study. The study analyzed data of patients treated between 1997 and 2011, retrieved from Taiwan's National Health Insurance database. We enrolled 32,801 patients who received coprescription of statins and CCBs that inhibit CYP3A4 (amlodipine, diltiazem, felodipine nicardipine, nifedipine, and verapamil). These patients were divided into 2 groups, according to whether they had received CYP3A4-metabolized statins (lovastatin, simvastatin, and atorvastatin) or non-CYP3A4-metabolized statins (fluvastatin, rosuvastatin, and pitavastatin). These 2 groups were 1:1 matched by age, gender, and Carlson comorbidity index. All outcomes were assessed within 90 days following drug coprescription.

In this study, 5857 patients received coprescription of CYP3A4-metabolized statins and CCBs that inhibit CYP3A4. There were no differences in comorbidity or use of antihypertensive drugs between patients who received CYP3A4-metabolized statins and those who received non-CYP3A4-metabolized statins. Patients who received CYP3A4-metabolized statins had significantly higher risk of acute kidney injury (adjusted odds ratio [OR] = 2.12; 95% CI = 1.35–3.35), hyperkalemia (adjusted OR = 2.94; 95% CI = 1.36–6.35), acute myocardial infarction (adjusted OR = 1.55; 95% CI = 1.16–2.07), and acute ischemic stroke (adjusted OR = 1.35; 95% CI = 1.08–1.68) than those who received non-CYP3A4-metabolized statins.

This nationwide cohort study demonstrated the increased risk of adverse events following the coprescription of CYP3A4-metabolized statins and CCBs that inhibit CYP3A4. Therefore, it is important to take into account the potential adverse events while coprescribing CYP3A4-metabolized statins and CCBs that inhibit CYP3A4.

## INTRODUCTION

Lipid-lowing drugs, particular statins, have been used worldwide to reduce the risk of cardiovascular events and death. Statin could be divided into 2 categories based on their metabolic pathway, cytochrome P450 3A4 (CYP3A4)-dependent and CYP3A4-independent. Based on pharmacokinetic characteristics, simvastatin, lovastatin, and atorvastatin are classified as CYP3A4-metabolized statins, while fluvastatin, rosuvastatin, and pitavastatin are non-CYP3A4-metabolized statins.^1^

Inhibitors of CYP3A4 could reduce presystemic metabolism of CYP3A4-metabolized statins and increase their plasma concentrations.^1^ Therefore, CYP3A4 inhibitors such as macrolide antibiotics frequently result in drug interactions with statins.^1,2^ It has been reported that coprescription of macrolide antibiotics with CYP3A4-metabolized statins increases the risk of statin toxicity, such as acute kidney injury and hyperkalemia.^3^ These effects were acute and could be observed within 30 days of coprescription.

Calcium channel blockers (CCBs) are one of the most popular drugs for hypertension. Certain CCBs, such as amlodipine, diltiazem, felodipine nicardipine, nifedipine, and verapamil, are relatively potent CYP3A4 inhibitors at clinically relevant dose.^4^ Drug–drug interactions could result after coprescription of CYP3A4-metabolized statins and CCBs that inhibit CYP3A4.

To the best of our knowledge, the potential risk of adverse events following the coprescription of statins and CCBs has been rarely reported. Therefore, we conducted a national, retrospective, and observational study to identify the adverse events after the coprescription of CYP3A4-metabolized statins and CCBs that inhibit CYP3A4.

## METHODS

### Data Collection

Data of patients who received statins between January 1997 and December 2011 were obtained from Taiwan's Longitudinal Health Insurance Database. The Longitudinal Health Insurance Database contains all of the registration files and details about the original claims that relates to 1 million beneficiaries from the National Health Insurance (NHI) database for research purposes. The NHI database holds information regarding outpatient data, inpatient data, disease profiles, the drugs prescribed, the intervention procedures, and the medical costs for more than 99% of the population in Taiwan, which equates to more than 22 million people. The diagnosis codes are based on the 9th revision of the International Classification of Diseases. To protect privacy, the individuals’ identifications are encrypted within the NHI database. This study was exempted from review by the Taipei Tzu Chi Hospital Review Board (IRB number: 03-W02-091).

### Study Population

**FIGURE 1:**
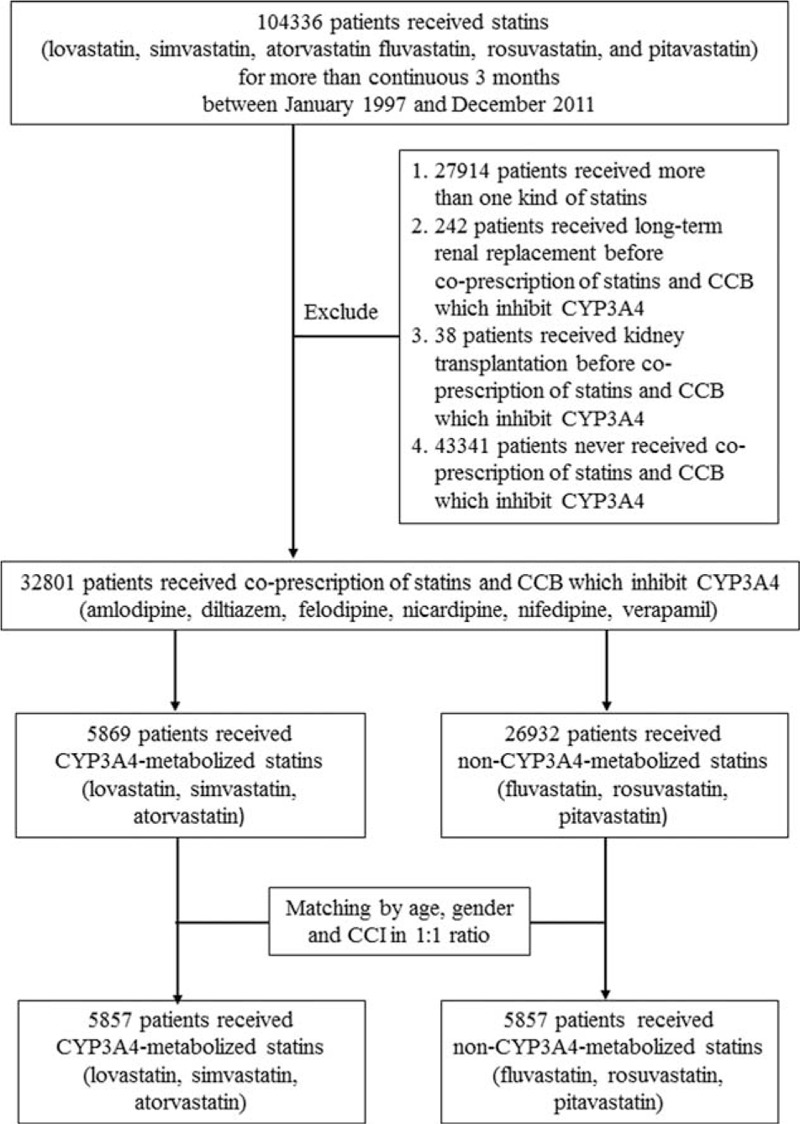
Flowchart of the study. CYP3A4 = cytochrome P450 3A4.

This was a population-based, longitudinal cohort study. Figure 1 illustrates the study subject selection process. Patients who received statins including lovastatin, simvastatin, atorvastatin fluvastatin, rosuvastatin, and pitavastatin for more than continuous 3 months between January 1997 and December 2011 were enrolled in the study. Patients who received more than one kind of statin, long-term renal replacement treatment, or kidney transplantation before receiving coprescription of statins and CCB which inhibit CYP3A4 were excluded from the study. Additionally, patients who never received coprescription of statins and CCB which inhibit CYP3A4 were excluded. Prescription of CCBs that inhibit CYP3A4 (amlodipine, diltiazem, felodipine nicardipine, nifedipine, and verapamil) within 30 days of receiving statin prescription was defined as coprescription. The patients who received coprescription of statin and CCBs were grouped according to whether they had received CYP3A4-metabolized statins (lovastatin, simvastatin, and atorvastatin) or non-CYP3A4-metabolized statins (fluvastatin, rosuvastatin, and pitavastatin). The 2 groups were 1:1 matched by age, gender, and Carlson comorbidity index. Baseline comorbidities were identified by ICD-9 codes, which included all cancers (140–172.9, 174–195.8), chronic kidney disease (582–582.9, 583–583.7, 585, 586, 588–588.9), coronary artery disease (414), diabetic mellitus (250–250.3, 250.7, 250.4–250.6), congestive heart failure (428–428.9), peripheral vascular disease (433.9, 411, 411.9, 785.4, V43.4), and cerebrovascular disease (430–437).

### Measurements of Outcomes

All outcomes were assessed within 90 days after the coprescription of statin and CCBs. We used ICD 9 codes for identifying adverse events including acute kidney injury (584), hyperkalemia (276.7), acute liver failure (570), acute myocardial infarction (410), and acute ischemic stroke (433, 434, 436) after coprescription. Furthermore, we compared 90 days outcomes in a restricted cohort at 3 periods: 90 days prior to coprescription, 90 days after coprescription, and 91 to 180 days after coprescription.

### Statistical Analysis

The patients’ baseline characteristics were compared using standardized difference, which has been used in previous studies.^5,6^ Standardized difference is less sensitive to sample size than traditional hypothesis test.^7^ They provided a measurement of the differences between groups divided by the pooled standard deviation. A value greater than 10% is interpreted as significant difference between the groups. In addition to the group effect, we adjusted for 13 potential confounding variables based on clinical relevance: age, sex, Charlson comorbidity score; baseline evidence of major cancers, renal disease, coronary artery disease, diabetic mellitus, congestive heart failure, peripheral vascular disease, cerebrovascular disease; baseline use of angiotensin-converting-enzyme inhibitors or angiotensin II receptor blockers, β-blockers, and diuretics. Univariate and multivariate logistic regression analyses were used to estimate odds ratios (ORs) and 95% confidence intervals (95% CIs) for all outcomes. All variants with significant difference (*P* value <0.05) in univariate analysis were entered into the multivariate model. In outcomes analysis, *P* values lower than 0.05 were interpreted as statistically significant. All of the statistical analyses were performed using SAS version 9.3 for Windows (SAS Institute, Inc., Cary, NC).

## RESULTS

In this study, 32,801 patients who received coprescription of statins and CCBs that inhibit CYP3A4 between January 1997 and December 2011 were enrolled. After grouping and matching, there were 5857 patients who received coprescription of CYP3A4-metabolized statins and CCBs that inhibit CYP3A4 (Figure 1). There were no differences in comorbidity or antihypertensive drugs between patients who received CYP3A4-metabolized statins and those who received non-CYP3A4-metabolized statins (Table 1).

**TABLE 1:**
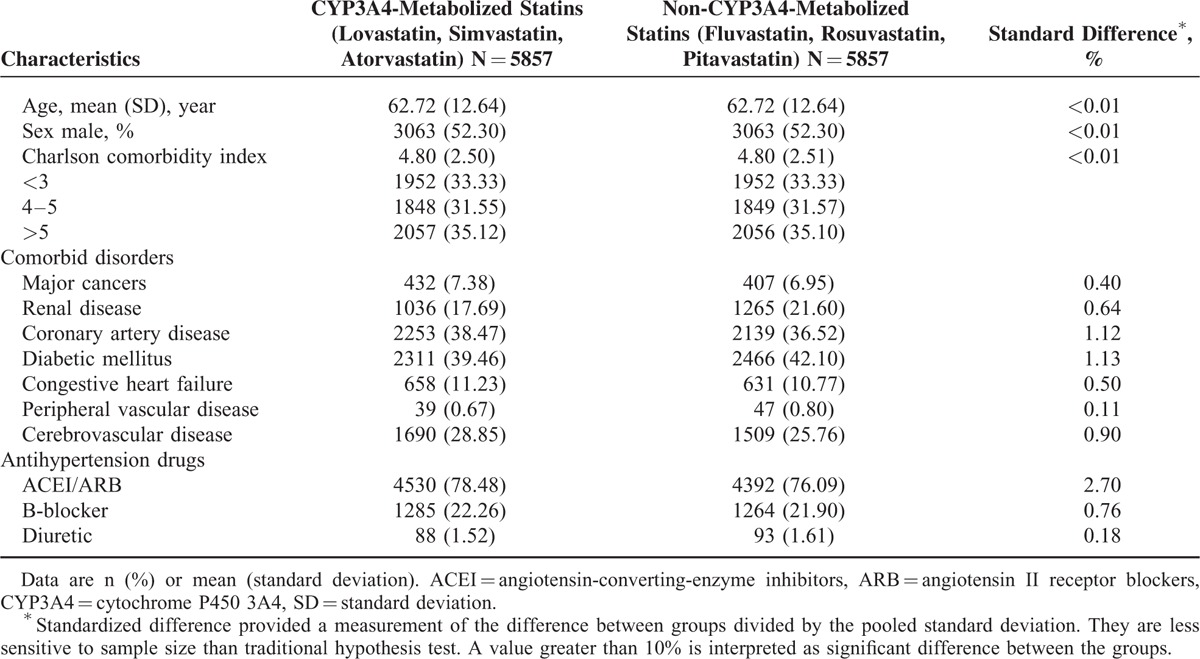
Baseline Characteristics

Results of all the outcomes within 90 days after coprescription are presented in Table 2. Patients who received CYP3A4-metabolized statins had significantly higher risk of acute kidney injury (adjusted OR = 2.12; 95% CI = 1.35–3.35), hyperkalemia (adjusted OR = 2.94; 95% CI = 1.36–6.35), acute myocardial infarction (adjusted OR = 1.55; 95% CI = 1.16–2.07), and acute ischemic stroke (adjusted OR = 1.35; 95% CI = 1.08–1.68) than those who received non-CYP3A4-metabolized statins. However, risk of acute liver failure or mortality between these 2 groups did not reach statistical significance.

**TABLE 2:**
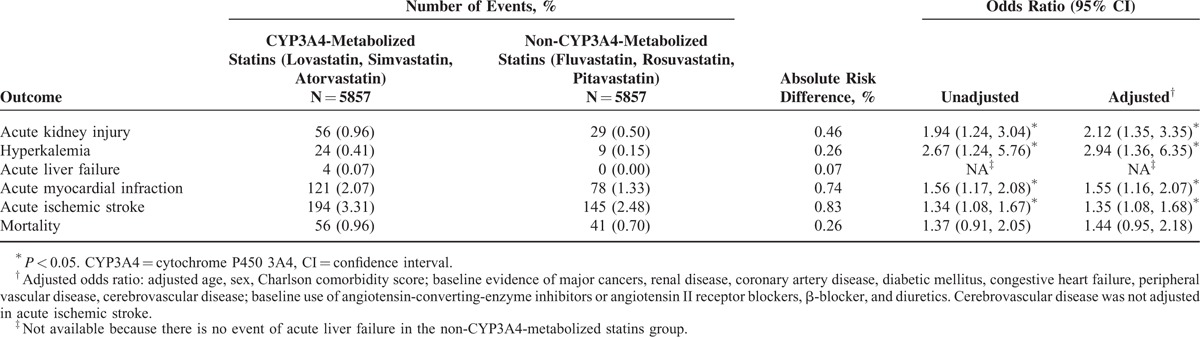
Risk of Adverse Events Within 90 days After Coprescription of Statins and Calcium Channel Blockers

In this cohort, univariate and multivariate logistic regression model showed the relationships between adverse events and baseline characteristics (Table 3). These results demonstrated that coprescription of CYP3A4-metabolized statin and CCBs that inhibit CYP3A4 independently contributed to acute kidney injury, hyperkalemia, acute myocardial infarction, and acute ischemic stroke.

**TABLE 3:**
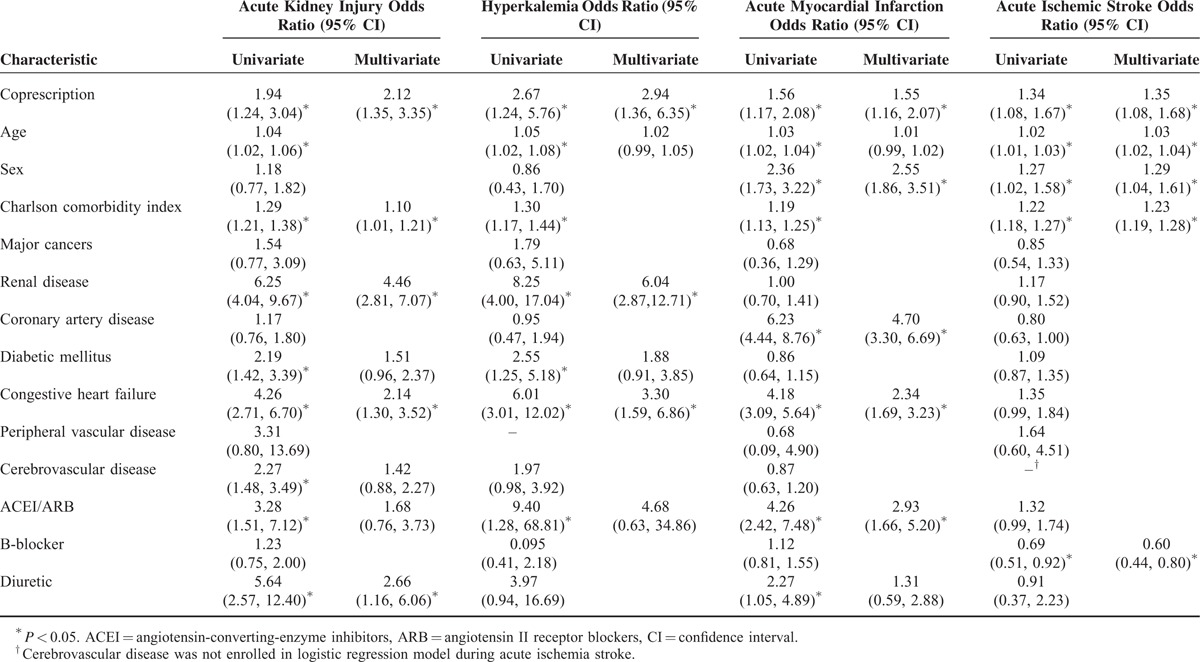
Univariate and Multivariate Logistic Regression Model Showing the Relationship between Risk of Adverse Events and Baseline Characteristics

Table 4 shows the risk of adverse events at different time periods, 90 days prior to coprescription and 91 to 180 days after coprescription of statin and CCBs. No significant differences of adverse events between CYP3A4-metabolized statins and non-CYP3A4-metabolized statins were observed at the time periods of either before or after coprescription of statins with CCBs that inhibit CYP3A4.

**TABLE 4:**
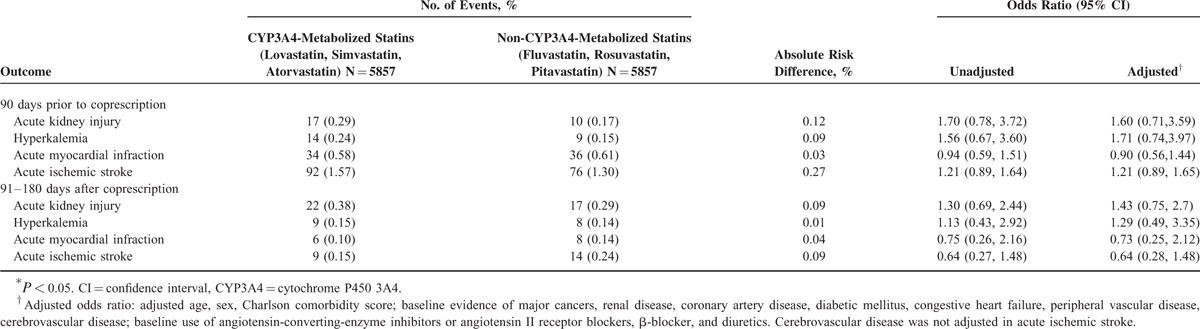
Risk of Adverse Events at Other Time Periods: 90 Days Prior to Coprescription and 91 to 180 Days After Coprescription of Statins and Calcium Channel Blockers That Inhibit CYP3A4

## DISCUSSION

The findings of our study demonstrated an increased risk of acute kidney injury, hyperkalemia, acute myocardial infarction, and acute ischemic stroke following the coprescription of CYP3A4-metabolized statins and CCBs that inhibit CYP3A4. To the best of our knowledge, this is the first population-based study to assess the adverse events of drug–drug interaction of CYP3A4-metabolized statins and CCBs that inhibit CYP3A4.

CYP3A4 is the one of the most abundant CYP enzyme in the liver and gut, and catalyzes the biotransformation of many drugs via oxidation.^8^ A number of important drugs have been identified as “substrates” or “inhibitors” of CYP3A4.^8^ In a prevalence study in Australia, potentially inappropriate CYP substrate–inhibitor interactions were reported in 6.2% of patients.^9^ The most commonly prescribed combinations that involved CYP3A4 were statins and CCBs.^9^ Therefore, we focused on the adverse events following the coprescription of statins (CYP3A4 substrate) and CCBs (CYP3A4 inhibitor) in our study.

Many in vitro studies have investigated the drug–drug interactions of statins and CCBs.^10–13^ Of the CCBs, verapamil and diltiazem could increase the area under the curve of simvastatin and lovastatin by 3- to 8-folds.^10,12^ As a consequence, it is reasonable to hypothesize the toxicity of statin after coprescription of CYP3A4-metabolized statins and CCBs (CYP3A4 inhibitor). In the present study, we did not compare the risk of adverse events of statins/CCBs versus stains alone, because this analysis would meet cofounding by indication. Patients who received both statins and CCBs might be sicker than those who received statins alone. Therefore, we observed interaction risk by comparing CYP3A4-metabolized statins/CCBs against non-CYP3A4-metabolized statins/CCBs in the observational study.

Statin toxicity associated with drug interaction might involve renal, muscular, and hepatic adverse events. In a previous case report, rhabdomyolysis occurred following combined administration of CYP3A4-metabolized statins and diltiazem.^14^ Patel et al had reported that coprescription of CYP3A4-metabolized statins and CYP3A4 inhibitors increased the risk of rhabdomyolysis, acute kidney injury, and hyperkalemia.^3^ In this study, we had found a higher risk of acute kidney injury and hyperkalemia following the coprescription of CYP3A4-matabolized statins and CCB. However, rhabdomyolysis was not surveyed, because its diagnosis might be underestimated by the database codes.^3^ Furthermore, our results showed that several episodes of acute liver failure were reported in the group of CYP3A4-metabolized statins but the numbers were small and did not reach statistical difference.

In addition, coprescription of CYP3A4-metabolized statins and CCBs that inhibit CYP3A4 could be associated with hypotensive complications. These adverse events might be due to a complex drug–drug interaction through CYP3A4. In addition to being “inhibitors” of CYP3A4, CCBs were also “substrates” that were metabolized by CYP3A4.^15^ Meanwhile, CYP3A4-metabolized statins were also “inhibitors” of CYP3A4.^1^ Therefore, coprescription of CYP3A4-metabolized statins with CCBs might have had a synergistic effect on CYP3A4 inhibition and could have interfered with the metabolism of CCBs. Therefore, there is the possibility of excessive systemic CCB concentration after coprescription. Gandhi et al^5^ had reported acute kidney injury and hypotension due to CCB toxicity when CYP3A4 was inhibited. In this study, the coprescription of CYP3A4-metabolized statins and CCBs. Therefore, the plasma CCBs levels were increased and resulted in hypotension and then to increase the risk of acute myocardial infarction and acute ischemic stroke. In our study, we examined the adverse events after coprescription of CYP3A4-matabolized statins and CCBs. The finding of acute kidney injury, acute myocardial infarction, and acute ischemic stroke might be associated with a hypotensive effect after coprescription. Further studies in vivo and in vitro are required to investigate the mechanism of drug–drug interactions of statins and CCBs.

In the present study, no significant differences of adverse events were observed between CYP3A4-metabolized statins and non-CYP3A4 metabolized statins when statins were prescribed without CCB (90 days prior to coprescription). The results implied that the risk of adverse events is attributed to coprescription rather than the statins itself. Furthermore, the risk of adverse events decreased significantly at later time period (91–180 days after coprescription) while compared with the 90 days after coprescription of statins with CCBs that inhibit CYP3A4. These results supported the fact that the risks of adverse events were obvious at the time of coprescription. Besides, drug modification by discontinuing statins or CCBs could occur after acute kidney injury or other complications.

Although the absolute risk difference was small, the outcomes in our study have important clinical implications. First, risks of adverse events after coprescription of statins and CCB might be preventable. When patients need the coprescription of statin and CCB, physicians could choose non-CYP3A4-metabolized statins rather than CYP3A4-metabolized statins to minimize the risks of adverse events. Second, this study could arouse researchers more attention to under-recognized drug–drug interactions. When patients received increased the number of different categories of prescription medications, the risk of drug–drug interactions might increase. More population-based studies and randomized controlled trials are necessary to confirm potential complications of drug–drug interactions. An effort should be made to minimize these preventable adverse events. Software support systems could be designed to identify CYP450 drug–drug interactions.^16,17^ Therefore, physicians who prescribe medications could receive warnings from electronic supportive system and avoid the potential risk of drug interactions.^18^

The present study has several limitations. First, we were unable to obtain information from the NHI database regarding the patients’ body height, body weight, and personal habits (physical activity, lifestyle, smoking, or alcohol consumption) in the NHIRD database. Second, statins and CCBs blood levels after coprescription or other laboratory data were not available in this database. Third, this was an observational study; hence, we could not be entirely certain that these associations of adverse events could be attributed to inhibition of CYP3A4 metabolism. Finally, whether the findings from this study could apply to other racial is uncertain because the majority of population in this study is Chinese ethnicity.

Aside from its shortcomings, this study has some strengths. The data employed in this study was obtained from the NHI research database, which covers most inpatient and outpatient medical practices for Taiwan's 23 million residents. It is one of the largest databases worldwide and has been used in many observational studies.^19–26^ In the present study, we compared CYP3A4-metabolized statins with non-CYP3A4-metabolized statins in order to minimize confounding by indication. In addition, we have minimized selection bias by matching control group with age, sex, and Charlson comorbidity score. Finally, we also verified that prescription of statins alone (90 days prior to coprescription) did not result in any statistically significant adverse events. This information reinforces the risk of coprescription of statins and CCBs.

In conclusion, our study found the associated risks of acute kidney injury, hyperkalemia, acute myocardial infarction, and acute ischemic stroke were higher following the coprescription of CYP3A4-metabolized statins and CCBs that inhibit CYP3A4. Further, the risks were lower in the coprescription of non-CYP3A4-metabolized statins and CCBs. Therefore, clinicians could take into account the potential adverse events during the coprescription of CYP3A4-metaboliized statins and CCBs that inhibit CYP3A4.
